# Estimating Long-Term Survival Temperatures at the Assemblage Level in the Marine Environment: Towards Macrophysiology

**DOI:** 10.1371/journal.pone.0034655

**Published:** 2012-04-11

**Authors:** Joëlle Richard, Simon Anthony Morley, Michael A. S. Thorne, Lloyd Samuel Peck

**Affiliations:** British Antarctic Survey, High Cross, Cambridge, United Kingdom; University of British Columbia, Canada

## Abstract

Defining ecologically relevant upper temperature limits of species is important in the context of environmental change. The approach used in the present paper estimates the relationship between rates of temperature change and upper temperature limits for survival in order to evaluate the maximum long-term survival temperature (Ts). This new approach integrates both the exposure time and the exposure temperature in the evaluation of temperature limits. Using data previously published for different temperate and Antarctic marine environments, we calculated Ts in each environment, which allowed us to calculate a new index: the Warming Allowance (WA). This index is defined as the maximum environmental temperature increase which an ectotherm in a given environment can tolerate, possibly with a decrease in performance but without endangering survival over seasonal or lifetime time-scales. It is calculated as the difference between maximum long-term survival temperature (Ts) and mean maximum habitat temperature. It provides a measure of how close a species, assemblage or fauna are living to their temperature limits for long-term survival and hence their vulnerability to environmental warming. In contrast to data for terrestrial environments showing that warming tolerance increases with latitude, results here for marine environments show a less clear pattern as the smallest WA value was for the Peru upwelling system. The method applied here, relating upper temperature limits to rate of experimental warming, has potential for wide application in the identification of faunas with little capacity to survive environmental warming.

## Introduction

Understanding the factors shaping variation in the physiology, ecology, and evolution of organisms is a fundamental issue for biologists [1]–[5]. Among environmental parameters temperature is possibly the best characterised and most fundamental as it affects the rates of all biochemical reactions; it drives species distribution and ecosystem level responses to climate variability [6]–[7]. In the context of environmental change, it is important to be able to evaluate not only the temperature limits of individual species but how this extends to the assemblage and ecosystem level across environments. Two main approaches currently dominate the investigation of likely responses of organism distributions to this change. The first is based on observations centred on identifying species ranges and comparing these with current and past environmental conditions to predict survival capabilities [8]–[10]. The second is based around experiments where animals are held in the laboratory or modified field situations and then conditions are manipulated [11]–[13]. Data from the latter will be utilised in this analysis.

Thermal tolerance is usually quantified with two different experimental methods [14], [15]: *i*) the *dynamic* method, which involves increasing or decreasing the test temperatures until an end point (e.g. the thermal point at which locomotory activity becomes disorganized and the animal loses its ability to escape from conditions that will promptly lead to its death) is reached and *ii*) the *static* method, which measures the time to death at constant test temperatures. In the description of these two types of experiment either the temperature exposure or time exposure are used to determine species tolerances. Salt [16], using experiments on insect cold-hardiness, was the first to take into account this duality by describing the log-linear relationship between the number of unfrozen insects and the time spent at different temperatures. Furthermore, using a mathematical model, Kilgour & McCauley [17] reconciled the two approaches by producing a method to transform data from one type of experiment to the other. In the same vein, Peck et al. [18] recognised the importance of both time and temperature in the establishment of temperature limits, when they evaluated upper temperature limits of Antarctic marine species by describing the relationship between rates of temperature change and upper temperature limits (maximum temperatures for survival) of a range of species.

In this paper, by combining the approach of Peck et al. [18] with the Kilgour & McCauley [17] model, data from the literature for both *dynamic* and *static* methods are used to evaluate subtidal marine species' upper temperature limits from several different temperate geographic regions. These are then discussed from a macrophysiological standpoint and compared with Antarctic data from Peck et al. [18]. Analysis of the relationship between upper temperature limits and the rates of temperature change allows an estimation of the maximum temperature at which a species can survive for several months, the long-term survival temperature (Ts). To place this new estimation of temperature limit in the context of a thermal tolerance threshold, it can be viewed as a temperature that is separate from the critical temperature (Tc) and the pejus temperature (Tp) defined by Pörtner [19]: *i*) Tc is the temperature at which the aerobic scope becomes minimal and the animal will begin to utilise anaerobic metabolic pathways, *ii*) Tp is the temperature at which performance falls below its optimum and correlations suggest that beyond this temperature, both abundance and growth begin to fall [20]. Ts, here, is defined as the temperature at which the capacity of the organism is not at its optimum but long-term survival is not threatened. Our approach allows us to evaluate how long-term survival thermal thresholds vary across widely separated marine ecosystems on a global scale, in relation to the temperature of the environment.

Moreover, in the context of climate warming, it becomes very important to be able to evaluate the physiological sensitivity of organisms to changes in the temperature of their environment [21]–[22]. With this aim, Deutsch et al. [23] used two heuristic indicators. Warming Tolerance, which is the difference between the critical thermal maximum of an ectotherm and the current climatological temperature of the organism's habitat (T_hab_, annual mean temperature). This quantity approximates the average amount of environmental warming an ectotherm can tolerate before performance drops to fatal levels. The second indicator is the Thermal Safety Margin which measures the difference between an organism's thermal optimum and its current climate temperature.

In this framework, two hypotheses can be tested: *i*) Adaptation to the cold always reduces the potential to cope with warm temperatures or, in other words, upper thermal tolerance decreases with habitat temperature *ii*) Warming tolerance increases with latitude [23]. To investigate the second hypothesis, a new index was used integrating the maximum long-term survival temperature. This index, called Warming Allowance (WA), approximates the maximum environmental temperature increase an ectotherm can tolerate with or without a decrease in performance but not endangering long-term survival. In this study, WA is used to evaluate the sensitivity of assemblages in different environments to warming.

## Materials and Methods

### Literature review

Data used in the analyses in this paper were collated from the published literature. For the overall approach comparing different temperate regions, the literature review focused on papers providing data on upper temperature limits and matching different criteria: *i*) marine subtidal species in temperate environments, *ii*) experiments conducted during the summer season and *iii*) experiments using preacclimation were included only if they were done at the corresponding *in situ* temperature. Two types of experiments were taken into account: *i*) those that used the *dynamic* method in which the temperature is progressively increased until 50% of the individuals in the trial have died (called LT_50_ by Stillman and Somero [24], used as upper thermal tolerance) and *ii*) the *static* method in which the temperature is raised to a set value and the time at which 50% of the individuals have died is recorded (measure of upper lethal temperature limits, ULT_50_) [14]. Six studies matched the criteria listed above [25]–[30], covering three taxonomic groups from different regions: Northern Hemisphere Warm Temperate environments (NHWT; South of France and West coast of the United States of America), Southern Hemisphere Warm Temperate environment (SHWT; Peru) and Cold Temperate environment (CT; West coast of Scotland; Table S1). The temperatures characterizing the different regions are presented in Table S3. In Peru, the recorded difference between the mean temperature in summer (15.8±1.2°C) and the mean temperature in winter (14.6±0.7°C) was only 1.2°C. Because the difference is small and not significant, we decided to include all the species in Urban [25], which included both summer and winter experiments. Taxonomic groups were pooled in this study to provide an assemblage level assessment. Peck et al. [18] in their inter-species upper temperature limits comparison tested for, but found no statistically significant effect of phylum. A similar analysis was considered here for tropical species but insufficient data were available in the literature.

Data were also available for four Mediterranean bivalve species [28]–[29] across seasons and they were therefore used to examine variability between seasons.

### Data transformation

Data from studies using the *static* method to assess upper lethal temperature limits (ULT_50_) had to be transformed in order to combine them with data from investigations using the *dynamic* method. The establishment of the measure of this limit provided by pooling data from the two kinds of experiments will be called here the upper temperature limit (UTL). The model used for this transformation was specifically designed to reconcile these two methods of measuring upper temperature limits [17]. The model is described in detail by Kilgour and McCauley [17], the following section describes only the major steps of the transformation used to allow data points from static temperature limit trials to be turned into dynamic temperature limit data points by calculating a rate of change.

K, b and T_c_ were constants found by fitting an exponential relationship to the data for time to 50% mortality (T_d_) versus each temperature (T) tested using the *static* method for each species.
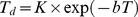
(1)T_c_ was determined graphically (Figure S1).

The rate of change was then calculated, by using these constants in the following equation:
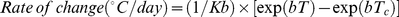
(2)This was the transformation used to compare short term, acute heating experiments with slow warming data throughout this investigation.

### Analysis

To compare linear regressions established from the log-log relationships of upper temperature limits versus rate of temperature change between each environment and between seasons, covariance analyses (ANCOVA) were used to test for differences in slope and intercept (if slopes were not significantly different).

For the macrophysiological analysis, the overall comparison of the different regions in the discussion, the maximum long-term survival temperature was estimated. A non linear model was fitted for the relationship between the upper temperature limit (UTL) and the rate of temperature change. Two standard forms of exponential models were fitted and the Aikaike Information Criterion (AIC) was used to select the best one. The first model tested followed this equation:
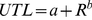
(3)and the second followed this equation:
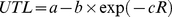
(4)Where UTL is the temperature at which 50% mortality occurred, R is the rate of temperature change expressed in day per °C and a, b and c are constants fitted by the model. The constant “a” was obtained as the asymptote of the curve and represented the maximum long-term survival temperature. The best model in the set used has the lowest AIC which was the second model (equation 4) for four of the five environments tested with ΔAIC (model AIC minus that of the best-fit model) values between 2.8 and 10.6. The only environment for which the AIC value was lower with the first model was the Northern Hemisphere Warm Temperate Shallow water environment with ΔAIC value of 3.8. In the discussion, only the second model is presented.

## Results

**Figure 1:**
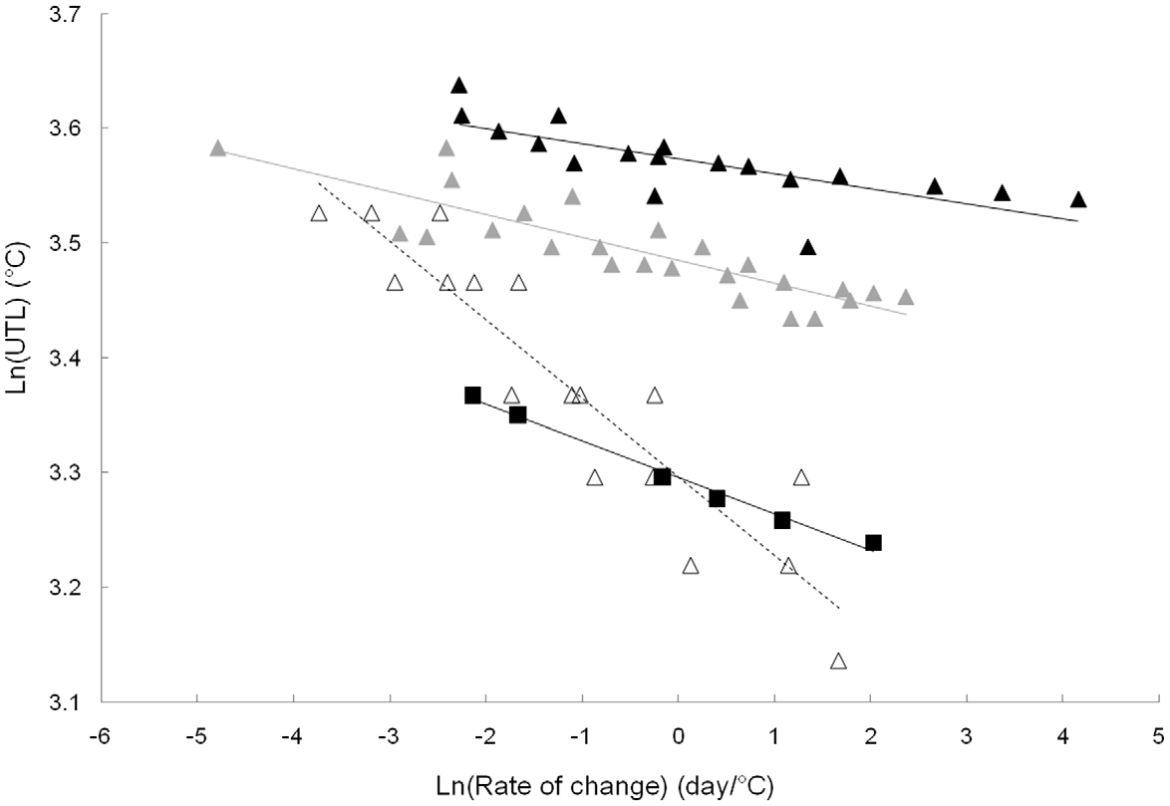
Log-log relationship for upper temperature limit (UTL) versus rate of temperature change. Only marine subtidal species from experiments done in summer are used in this figure. Data for the *dynamic method* were combined with those from the *static method* after transformation using the Kilgour and McCauley [17] model. Black triangles: species from Northern Hemisphere Warm Temperate environments (NHWTSW) in the South of France and the West coast of the United States of America in very Shallow Water (<2 m); grey triangles: species from Northern Hemisphere Warm Temperate environments (NHWT) in the South of France and the West coast of the United States of America in deeper water (>2 m); open triangles: species from a Southern Hemisphere Warm Temperate environment (SHWT) on the Peru coast; and black squares: species from a Cold Temperate environment (CT) in Scotland. For each ecosystem, a linear regression is fitted. Black triangles: y = −0.01×+3.6 R^2^ = 0.57, grey triangles: y = −0.02×+3.5 R^2^ = 0.75, open triangles: y = −0.07×+3.3 R^2^ = 0.86, black squares: y = −0.03×+3.3 R^2^ = 0.99.

The log-log relationship for upper temperature limits of temperate marine species versus rate of temperature change showed different patterns between regional groups (Figure 1, Table 1). Based on the covariance analysis (Table 1), the group of species from the Northern Hemisphere Warm Temperate environment was further split in two: one with shallow water species (<2 m) and the other with species from slightly deeper sites, with the intercepts of the slopes being different. This lead to four groups being identified: *i*) species from Northern Hemisphere Warm Temperate environments in shallow water (NHWTSW), *ii*) species from Northern Hemisphere Warm Temperate environments beyond 2 m depth (NHWT), *iii*) species from Southern Hemisphere Warm Temperate environment (SHWT) and *iv*) species from Cold Temperate environment (CT). In each group, the upper temperature limit decreased at slower rates of temperature change. There was also a lowering of the intercepts of the regression lines from the NHWTSW to the CT environment. Furthermore, the intercepts were directly linearly related to the maximum habitat temperature (Tmax) of the group's location (0.03Tmax+2.88; p = 0.02). For the Northern hemisphere groups, the slope of the linear regression increased slightly (in absolute value) from NHWTSW to CT environments. The Southern hemisphere group differed from all others as it had a steeper slope.

**Table 1:** Results of covariance analysis corresponding to the linear regressions drawn in Figure 1 plus Antarctic data.

Intercept*Slope*	*NHWTSW*	*NHWT*	*SHWT*	*CT*	*A*
***NHWTSW***		<0.0001*			
***NHWT***	*0.07*			<0.0001*	
***SHWT***	*<0.0001* *	*<0.0001* *			<0.0001*
***CT***	*0.01* *	*0.06*	*0.013* *		<0.0001*
***A***	*0.01* *	*0.004* *	*0.27*	*0.37*	

P-values are indicated for the comparison between slopes and intercepts for each pair of environments. NHWTSH: Northern Hemisphere Warm Temperate environments in Shallow Water (<2 m), NHWT: Northern Hemisphere Warm Temperate environments in deeper water, SHWT: Southern Hemisphere Warm Temperate environment, CT: Cold Temperate environment and A: Antarctic environment.

* : statistically significant.

**Figure 2:**
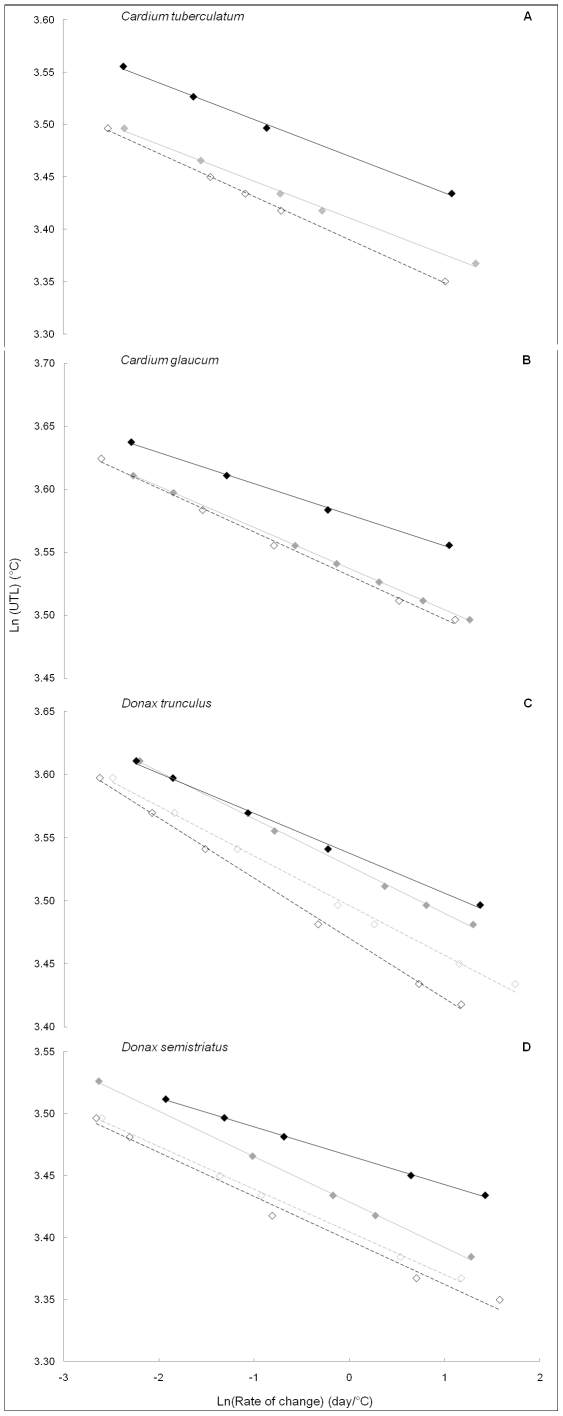
Log-log relationship for upper temperature limit (UTL) versus rate of temperature change. This is shown for four different species from the Mediterranean Sea in different seasons: A) *Cardium tuberculatum*, B) *Cardium glaucum*, C) *Donax trunculus* and D) *Donax semistriatus*. Black diamonds: summer, open black diamonds: winter, grey diamonds in a and b: autumn and spring, grey diamonds in c and d: autumn and open grey diamonds: spring. For each season and each species a linear regression is fitted. A) Black diamonds: y = −0.034×+3.5 R^2^ = 1, grey diamonds: y = −0.034×+3.4 R^2^ = 1, open black diamonds: y = −0.04×+3.4 R^2^ = 1; B) Black diamonds: y = −0.024×+3.6 R^2^ = 1, grey diamonds: y = −0.032×+3.5 R^2^ = 1, open black diamonds: y = −0.034×+3.5 R^2^ = 1; C) Black diamonds: y = −0.032×+3.5 R^2^ = 1, grey diamonds: y = −0.038×+3.5 R^2^ = 1, open grey diamonds: y = −0.039×+3.5 R^2^ = 1, open black diamonds: y = −0.048×+3.5 R^2^ = 1; D) Black diamonds: y = −0.023×+3.5 R^2^ = 1, grey diamonds: y = −0.037×+3.4 R^2^ = 1, open grey diamonds: y = −0.034×+3.4 R^2^ = 1, open black diamonds: y = −0.035×+3.4 R^2^ = 0.99.

For the Mediterranean species, where data were available for each season, relationships followed the same pattern; upper temperature limits decreased as rates of temperature change (experimental warming) decreased (Figure 2). For all species, a decrease in intercept was observed from summer to winter (i.e. from warmer to cooler periods), and this was coupled with an increase in slope (in absolute value; Tables 2 and 3).

**Table 2:** Results of covariance analysis corresponding to the linear regressions drawn in Figure 2 A and B.

Species	Intercept *Slope*	*Winter*	*Spring and Autumn*	*Summer*
***Cardium tuberculatum***	***Winter***			
	***Spring and Autumn***	*0.005* *		<0.0001*
	***Summer***	*0.007* *	*0.94*	
***Cardium glaucum***	***Winter***		0.008*	
	***Spring and Autumn***	*0.08*		
	***Summer***	*0.001* *	*<0.0001* *	

P-values are indicated for the comparison between slopes and intercepts for each season and species.

* : statistically significant.

**Table 3:** Results of covariance analysis corresponding to the linear regressions drawn in Figure 2 C and D.

Species	Intercept *Slope*	*Winter*	*Spring*	*Autumn*	*Summer*
***Donax trunculus***	***Winter***				
	***Spring***	*0.0004* *		<0.0001*	
	***Autumn***	*<0.0001* *	*0.24*		
	***Summer***	*<0.0001* *	*0.003* *	*0.002* *	
***Donax semistriatus***	***Winter***		0.16	<0.0001*	
	***Spring***	*0.71*		<0.0001*	
	***Autumn***	*0.64*	*0.9*		
	***Summer***	*0.003* *	*0.0001* *	*<0.0001* *	

P-values are indicated for the comparison between slopes and intercepts for each season and species.

* : statistically significant.

## Discussion

### Effect of varying rate of warming on upper thermal tolerance limits

Previously, Peck et al. [18] showed that upper temperature limits varied in a curvilinear fashion with different rates of warming for 14 species of Antarctic marine ectotherms.

In this study, similar relationships between the upper temperature limit and the rate of change of experimental temperature were obtained for marine subtidal temperate species acclimated to summer *in situ* temperatures. Four different groups were identified corresponding to the different regions. Due to significant differences in the relationship (Table 1) the group from the Northern Hemisphere Warm Temperate environment was split in two: one with species in very shallow water (less than two meters deep) and species in deeper water. This could be caused by the fact that temperature is slightly higher in very shallow water habitats (Table S3). At any rate of warming, the upper temperature limit decreased with habitat temperature across Northern hemisphere environments, which is consistent with Stillman and Somero [24], who are suggested that the ability of heat tolerance may be lost in absence of selection for it. The group from South America (SHWT) had different responses to elevated temperature than the other three, as the slope of the linear regression was steeper (Table 1).

### Macrophysiological analysis

**Figure 3:**
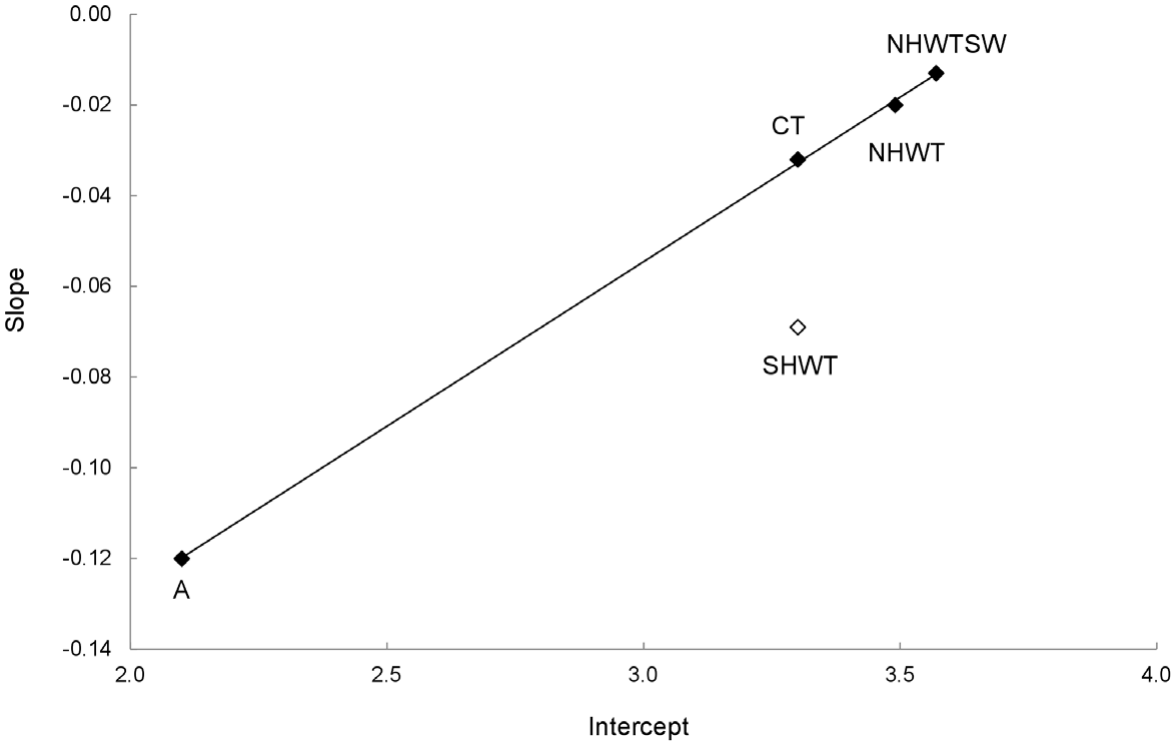
Correlation between Slope and Intercept (from **Figure 1** **+Antarctic environment).** Each diamond represents an environment. NHWTSW: Northern Hemisphere Warm Temperate environments in the South of France and the West coast of the United States of America in very Shallow Water (<2 m); NHWT: Northern Hemisphere Warm Temperate environments in the South of France and the West coast of the United States of America in deeper water (>2 m); SHWT: Southern Hemisphere Warm Temperate environment on the Peru coast; CT: Cold Temperate environment in Scotland; A: Antarctic environment. Linear regression SHWT excepted: y = 0.07×−0.27, R^2^ = 0.99, p-value: 0.0003.

The data presented here for species from temperate regions can be compared to those for benthic fish, bivalves and gastropods from Antarctica (A) from Peck et al. [18] (Table S2). The analysis was taxonomically restricted to minimise any added variability due to taxonomic differences. Based on the expanded data set (Figure 3, Table 1), slopes of the log-log relationships (slopes from the Figure 1+Antarctic environment) became steeper from the warmest (NHWTSW) to the coldest environments (A). Moreover, the intercept (I) was linearly correlated to the slope in this relationship when the SHWT was excluded (0.07I-0.27; p = 0.0003). Indeed, in this framework, the SHWT environment studied appears to be an exception, as the slope for this environment differed significantly from those of all the other temperate environments studied and was closer to that of the Antarctic environment (Table 1) than other temperate sites.

### How does adaptation to the cold reduce resistance to the warm?

Stillman and Somero [24] observed on a vertical gradient that although low-intertidal and subtidal species generally have LT_50_'s that are considerably higher than maximum microhabitat temperatures, the actual LT_50_'s are lower than those that ancestral, intertidal species may have had. From these observations, they suggested that, in the absence of selection for heat tolerance, this ability may be lost or that there may be physiological costs involved in maintaining an elevated LT_50_. Moreover, Pörtner [19], showed that critical temperatures (Tc) differ between species and populations depending on latitude or seasonal temperature acclimatisation and are therefore related to geographic distribution.

In this study, a significant trend in the slope of the relationship between upper temperature limits and rate of experimental warming across regions is observed as an overall lowering of the elevation of the relationships from warm to cold environments. This is in agreement with the hypothesis suggested by Stillman and Somero [24]. Moreover, this is mirrored by a similar trend for the data for seasonal effects on this relationship in bivalve molluscs from the Mediterranean Sea (Figure 2, Tables 2 and 3), where the intercepts of the relationships was lower in winter. The seasonal acclimatisation of upper thermal tolerance is also in agreement with Stillman and Somero [24] concerning the cost of maintaining an elevated upper temperature limit.

Upper temperature limits are lower in colder environments both at slow and fast rates of temperature change. This follows the paradigm that the more a species is adapted to cold temperatures, the lower its ability to survive high temperatures. However, the upwelling environment off Peru (SHWT) differs from the other environments, where the loss of ability to cope with high temperatures is not as marked. An explanation for this may be that in the Peru system, species with very different historical origins coexist. For example, *Argopecten purpuratus* is a relic of a tropical/subtropical fauna that once dominated the Peruvian shores during the Miocene [31] while the most recently colonizing macro benthic species of the area (e.g. the bivalve mollusc *Gari solida*) are more typical upwelling – adapted, cold – water species [32]. This environment is unusual in containing a mix of both species originally “warm-adapted” and species originally “cold-adapted”. This is in contrast with the other environments studied that generally contain only species coming historically from warmer environments. This may account for the unusual responses to rate of warming for the Peru upwelling fauna compared to the other regions studied, as demonstrated by the different slope in Table 1.

### Are species from some environments more vulnerable than others?

**Figure 4:**
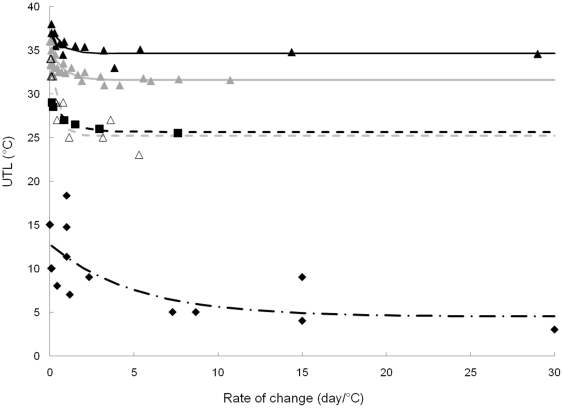
Correlation between upper temperature limit (UTL) and rate of temperature change. Black triangles: Northern Hemisphere Warm Temperate environments in Shallow Water (<2 m; NHWTSW), grey triangles: Northern Hemisphere Warm Temperate environments in deeper water (NHWT), open triangles: Southern Hemisphere Warm Temperate environment (SHWT), black squares: Cold Temperate environment (CT) and black diamonds: Antarctica (A). For each environment, the non linear model described in the methods is fitted.

A possible way to explain the translation in upper temperature limits between the five environments studied can be obtained by comparing species' upper temperature limits with the field summer temperature at each site. This was done by fitting a non linear model to the data of upper temperature limit versus rate of change for each environment (Figure 4). The value of the asymptote given by the model represents the maximum long-term survival temperature. For each environment studied, calculating long-term survival limits this way is more robust than a direct measure because it uses temperature limits across a wide range of warming scenarios and is affected less by short-term factors that may be important in single time point experiments. The maximum long-term survival temperature obtained can be compared with the *in situ* mean maximum observed habitat temperature to identify any possible relationship. The difference between the maximum long-term survival temperature and the *in situ* mean maximum temperature varies from 2.9 to 9.7 (Table 4). These values can be defined, at least in principle, as the maximum environmental temperature increase which species groups in each environment can tolerate (with or without a decrease in performance but not endangering survival). We can call this the Warming Allowance (WA). Compared to other indices used to evaluate sensitivity of the species to environmental warming, the WA was designed to be of more ecological relevance. The two other indices previously used utilized different temperature limits for comparison to habitat temperature [23]. The Thermal Safety margin (TSM) is calculated from the optimum temperature and the Warming Tolerance (WT), with the CTmax. The first one overrates the sensitivity of a species to environmental warming while the latter underestimates it.

**Table 4:** Maximum long-term survival temperature estimate from the asymptote value given by the non linear model and the standard error corresponding.

Environment	Maximum long-term survival temperature (°C)	Standard error	WA
***NHWTSW***	34.6	0.3	8.5
***NHWT***	31.6	0.3	6.2
***SHWT***	25.2	0.7	7.7
***SHWT out of El Niño event***	25.2	0.7	8.7
***SHWT during El Niño event***	25.2	0.7	2.9
***CT***	25.7	0.2	9.7
***A***	4.5	2.4	3.5

WA is the Warming allowance represented by the difference between the maximum long-term survival temperature and the *in situ* mean maximum temperature. NHWTSH: Northern Hemisphere Warm Temperate environment in Shallow Water (<2 m), NHWT: Northern Hemisphere Warm Temperate environment in deeper water, SHWT: Southern Hemisphere Warm Temperate environment, CT: Cold Temperate environment and A: Antarctic environment.

Using the new WA index to evaluate latitudinal patterns of the relationship between long-term survival limits and the maximum experienced environmental temperature for marine environments, produces a pattern that is not straightforward. This contrasts with previous studies that have found, or assumed, linear changes in thermal tolerance or acclimation capacity across latitudinal gradients e.g. [23], [33]–[34]. These studies also suggested the biological impacts of temperature change may be most profound in tropical rather than in temperate areas despite predictions from climate models for much smaller thermal changes in the former. In our study, the species where long-term survival limits were closest to experienced maximum temperatures were from the upwelling ecosystem in the South Pacific. The link between the WA and physical environmental characteristics can also be assessed in relation to environment temperature variability. Although the data are limited, and care is needed in interpretation, the calculated long-term survival limits from more variable environments are further above the experienced summer maxima than those from more stable regions, following the predictions of the climate variability hypothesis [35]. Thus the more variable temperature environments studied here are the cold temperate and the warm temperate sites in the North hemisphere. At these sites the WA values are between 6.2 and 9.7 while in the least variable region (Antarctica) the WA value is 3.5.

### Antarctic ecosystem

The WA value of the Antarctic environment is particularly low compared to temperate values with a maximum long-term survival temperature of 4.5°C. Long-term acclimation studies have been done on different species from Antarctica [18], [36] showing that several of the species tested were not able to survive one month at 4°C. These data corroborate our estimation of the maximum long-term survival temperature and agree with data showing that in highly stenothermal environments such as Antarctica, acclimation capacity is low [36].

### An upwelling ecosystem: a particular case to test the WA validity

The Peru upwelling ecosystem has a WA value similar to the other temperate regions, despite usually being described as an environment with little variation in annual water temperatures (between 16 and 21°C) [25]. However, this ecosystem is exposed intermittently, on average every 3 to 7 years, to the effects of the El Niño/Southern Oscillation (ENSO) which varies in intensity [37]. It leads to two changes which are fundamental for the Latin American Pacific ecosystem: water temperature increases and the thermocline drops deeper [25]. The mean maximum summer sea temperature during 1983–2004 which included two El Niño events (1982–1983 and 1997–1998) was 17.5°C. It was 16.5°C excluding the El Niño events but rose to 22.3°C during the El Niño years with peaks of more than 25°C [32]. Interestingly, when our extrapolated WA values are compared to habitat maxima during El Niño years, a low value (2.9) is obtained which could even be close to zero during extreme El Niño events. Our analysis thus indicates that the species living there are not able to cope with the high temperatures of El Niño events and can only survive short periods before dying during these events. Ecological studies confirm the negative impact of the El Niño events on these populations [37]–[38] with significant mortalities and large reductions in reproductive effort reported. Thus, if the variability of the environment is often linked with phenotypic plasticity [3], [5], [39], the unpredictability of the environment coupled with irregular extreme events such as El Niño can have substantial influences on survival [40]–[43].

### Conclusions

This study presents a new method for estimating the maximum long-term survival temperature, for a species, population or community which can be used to compare organisms from different environments and evaluate their sensitivity to environmental change. Moreover, this study shows that multiple factors have to be taken into account to understand the upper temperature limits of species in different environments, these include: *i*) the maximum temperature of the environment, *ii*) the temperature variability and predictability of the environment and *iii*) the evolutionary history of the species. Further questions need to be answered to increase understanding of the impact of environmental change. These include the link between the evolutionary history of a species and the potential of this species to adapt to change; are species more finely adapted to their environments less adaptable? One way to try to address this question could be by comparing species from Arctic, Antarctic and tropical environments which are all stable in terms of temperature, but which contain species with markedly different evolutionary histories.

## Supporting Information

Figure S1 **Additional information for the model used to transform the data.** Graphical illustration of the method used to obtain the different constants used in equation 2. The equation in the figure comes from fitting the exponential curve and the circles show the values needed to calculate rate of change with Kilgour & McCauley's model [17].
(TIFF)

Table S1 **Location, region, depth and type of experiment for temperate species used in the analysis.**
(DOC)

Table S2 **Location, region, depth and type of experiment for Antarctic species used in the analysis.**
(DOC)

Table S3 **Environmental parameters for the different sites studied.**
(DOC)
