# Supplementary figures and images for: Estimating Long-Term Survival Temperatures at the Assemblage Level in the Marine Environment: Towards Macrophysiology
Source: PLoS One. 2012 Apr 11;7(4):e34655. doi: 10.1371/journal.pone.0034655 (PMC3324497; doi:10.1371/journal.pone.0034655)

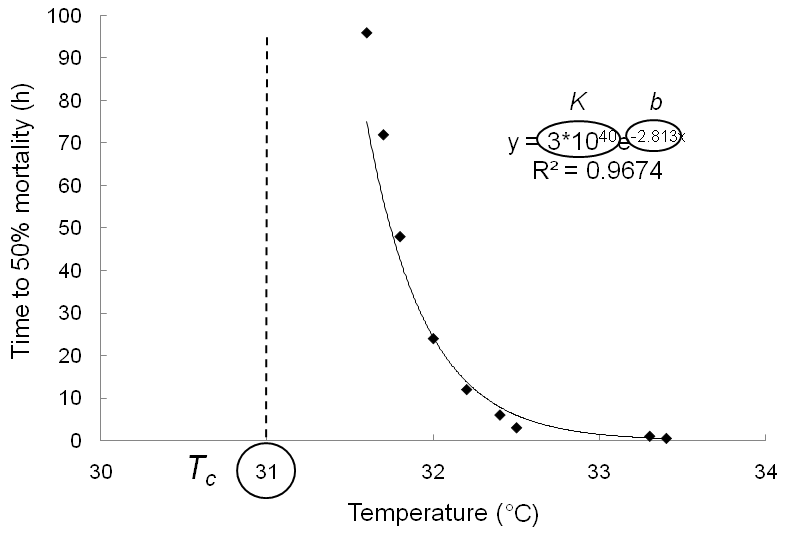

Supplement: Figure S1 — Additional information for the model used to transform the data. Graphical illustration of the method used to obtain the different constants used in equation 2. The equation in the figure comes from fitting the exponential curve and the circles show the values needed to calculate rate of change with Kilgour & McCauley's model [17]. (TIFF) [file pone.0034655.s001.tif]
