# Supplementary material for: Estimating Long-Term Survival Temperatures at the Assemblage Level in the Marine Environment: Towards Macrophysiology
Source: PLoS One. 2012 Apr 11;7(4):e34655. doi: 10.1371/journal.pone.0034655 (PMC3324497; doi:10.1371/journal.pone.0034655)
Supplement: Table S1 — Location, region, depth and type of experiment for temperate species used in the analysis. (DOC) [file pone.0034655.s002.doc]

**Table S1**: **Location, region, depth and type of experiment used to determine upper temperature limits of the species**.

| **Species** | **Location** | **Region** | **Depth** | **Data** | **Reference** |
| --- | --- | --- | --- | --- | --- |
| *Tellina tenuis*  (Bivalvia) | South of France  43°21’N;04°53’W | NHWT | 2-3m | S | [27] |
| *Donax semistriatus*  (Bivalvia) | South of France  43°21’N;04°53’W | NHWT | 4-5m | S | [28] |
| *Donax trunculus*  (Bivalvia) | South of France  43°21’N;04°53’W | NHWT | 0.5-1m | S | [28] |
| *Cardium glaucum*  (Bivalvia) | South of France  43°21’N;04°53’W | NHWT | 0.5-1m | S | [29] |
| *Cardium tuberculatum*  (Bivalvia) | South of France  43°15’N;05°22’W | NHWT | 4-5m | S | [29] |
| *Tegula montereyi*  (Gastropoda) | California  36°36'N;121°54'W | NHWT | 3-12m | D | [30] |
| *Fundulus parvipinnis*  (Fish) | California  32°47’N;117°13W | NHWT | Shallow water | S | [26] |
| *Atherinops affinis*  (Fish) | California  32°52’N;117°15’W | NHWT | Open water near shore | S | [26] |
| *Gari solida**  (Bivalvia) | Peru  14°15'S;76°10'W | SHWT | 2-16m | S | [25] |
| *Semele solida**  (Bivalvia) | Peru  14°15'S;76°10'W | SHWT | 2-16m | S | [25] |
| *Semele corrugate**  (Bivalvia) | Peru  14°15'S;76°10'W | SHWT | 2-16m | S | [25] |
| *Argopecten pupuratus*  (Bivalvia) | Peru  14°15'S;76°10'W | SHWT | 2-16m | S | [25] |
| *Tellina fabula*  (Bivalvia) | West coast Scotland  55°45’N;04°55’W | CT | 8-10m | S | [27] |

Location, region, depth and type of experiment used to determine upper temperature limits of the species from the different studies used in the analysis of effects of rate of temperature change on temperature limits. The place name indicates where the study was conducted. Missing location positions (latitude and longitude) were estimated using Google Earth^TM^. *: species for which data were collected in winter. NHWT: Northern Hemisphere Warm Temperate environment; SHWT: Southern Hemisphere Warm Temperate environment; CT: Cold Temperate environment. S: *Static* method; D: *Dynamic* method.
