# Supplementary material for: Estimating Long-Term Survival Temperatures at the Assemblage Level in the Marine Environment: Towards Macrophysiology
Source: PLoS One. 2012 Apr 11;7(4):e34655. doi: 10.1371/journal.pone.0034655 (PMC3324497; doi:10.1371/journal.pone.0034655)
Supplement: Table S2 — Location, region, depth and type of experiment for Antarctic species used in the analysis. (DOC) [file pone.0034655.s003.doc]

**Table S2: Location, region, depth and type of experiment for Antarctic species used in the analysis.**

| **Species** | **Location** | **Region** | **Depth** | **Data** | **Reference** |
| --- | --- | --- | --- | --- | --- |
| *Trematomus bernacchii*  (Fish) | Mc Murdo Sound  77°51’S;166°38’E | A | NA | S | [44][[1]](#footnote-2) |
| *Trematomus borchgrevinki*  (Fish) | Mc Murdo Sound  77°51’S;166°38’E | A | NA | S | [44] |
| *Trematomus hansoni*  (Fish) | Mc Murdo Sound  77°51’S;166°38’E | A | NA | S | [44] |
| *Laternula elliptica*  (Bivalvia) | Rothera  67°34’S;68°08’W | A | 5-25m | D | [18] |
| *Laternula elliptica*  (Bivalvia) | Rothera  67°34’S;68°08’W | A | 29-33m | D | [45][[2]](#footnote-3) |
| *Limopsis marionensis*  (Bivalvia) | Eastern Weddell sea  74°10’S;27°40’W | A | NA | D | [46][[3]](#footnote-4) |
| *Yoldia eightsi*  (Bivalvia) | Rothera  67°34’S;68°08’W | A | 5-25m | D | [18] |
| *Nacella concinna*  (Gastropoda) | Rothera  67°34’S;68°08’W | A | 5-25m | D | [18] |
| *Nacella concinna*  (Gastropoda) | Signy Island  60°72’S;45°60W | A | 5-15m | D | [47][[4]](#footnote-5) |

Location, region, depth and type of experiment used to determine upper temperature limits of the Antarctic species from the different studies used in the analysis of effects of rate of temperature change on temperature limits. The place name indicates where the study was conducted. Missing location positions (latitude and longitude) were estimated using Google Earth^TM^. A: Antarctica. S: *Static* method; D: *Dynamic* method. NA: not available.

1. 44. Somero GN, DeVries AL (1967) Temperature tolerance of some Antarctic fishes. Science 156: 257-258. [↑](#footnote-ref-2)
2. 45. Peck LS, Pörtner HO, Hardewig I (2002) Metabolic demand, oxygen supply, and critical temperatures in the Antarctic bivalve *Laternula elliptica*. Physiol Biochem Zool 75: 123-133. [↑](#footnote-ref-3)
3. 46. Pörtner HO, Peck LS, Zielinski S, Conway LZ (1999) Intracellular pH and energy metabolism in the highly stenothermal Antarctic bivalve *Limopsis marionensis* as a function of ambient temperature. Polar Biol 22: 17-30. [↑](#footnote-ref-4)
4. 47. Peck LS (1989) Temperature and basal metabolism in two Antarctic marine herbivores. J Exp Mar Biol Ecol 127: 1-12. [↑](#footnote-ref-5)
