# Supplementary material for: Estimating Long-Term Survival Temperatures at the Assemblage Level in the Marine Environment: Towards Macrophysiology
Source: PLoS One. 2012 Apr 11;7(4):e34655. doi: 10.1371/journal.pone.0034655 (PMC3324497; doi:10.1371/journal.pone.0034655)
Supplement: Table S3 — Environmental parameters for the different sites studied. (DOC) [file pone.0034655.s004.doc]

**Table S3:** **Environmental parameters for the different sites studied**.

| ***Environment*** | ***T_mean_*** | ***SD*** | ***T_mean_ for Summer month*** | ***SD*** | ***T_max_ for Summer month*** | ***SD*** |
| --- | --- | --- | --- | --- | --- | --- |
| NHWTSW | 18.5 | 4.3 | 24.2 | 1.6 | 26.2 | 1.0 |
| NHWT |  |  | 19.6 | 0.9 | 25.4 | 1.3 |
| SHWT | 15.4 | 1.9 | 16.4 | 2.2 | 17.5 | 2.7 |
| SHWT out of El Niño event | 15.0 | 1.1 | 15.9 | 1.3 | 16.5 | 1.5 |
| SHWT during El Niño event | 17.6 | 2.9 | 22.3 | 1.2 | 22.3 | 1.1 |
| CT | 10.9 | 2.5 | 14.1 | 1.1 | 16.0 | 1.1 |
| A | -0.7 | 0.9 | 0.1 | 0.7 | 1.0 | 0.7 |

Average annual, summer and maximum summer temperature and their standard deviation for the different regions used in this study. *In situ* temperature sources for the different ecosystems: 1) NHWTSW: seawater surface temperature in Frioul, Marseille from 1999 to 2008 (SOMLIT); 2) NHWT: temperature data for Riou Island (NW Mediterranean, France) from July to September from 1999 to 2006 at 10m depth [48][[1]](#footnote-2); 3) SHWT: temperature recorded at La Vieja Island (Independencia bay, Peru) by the Instituto del Mar del Peru (IMARPE) for Pisco during the period 1979 to 2001. Two El Niño warming events occurred during this period, the first in 1982-1983 and the second in 1997-1998; 4) CT: temperature recorded at Keppel Pier, Isle of Cumbrae, Firth of Clyde (Millport, Scotland) from 2004 to 2008 by the University Marine Biological Station, Millport; 5) A: temperature recorded at Rothera (Antarctic Peninsula) from 1999 to 2008 by the Rothera Oceanographic and Biological Time Series. NHWTSH: Northern Hemisphere Warm Temperate environments in Shallow Water (<2 m), NHWT: Northern Hemisphere Warm Temperate environments in deeper water, SHWT: Southern Hemisphere Warm Temperate environment, CT: Cold Temperate environment, A: Antarctic environment, SD: standard deviation.

1. 48. Torrents O, Tambutté E, Caminiti N, Garrabou J (2008) Upper thermal thresholds of shallow vs. deep populations of the precious Mediterranean red coral *Corallium rubrum* (L.): Assessing the potential effects of warming in the NW Mediterranean. J Exp Mar Biol Ecol 357: 7-19. [↑](#footnote-ref-2)
